# Intracellular Ca^2+^ signalling: unexpected new roles for the usual suspect

**DOI:** 10.3389/fphys.2023.1210085

**Published:** 2023-07-27

**Authors:** Francesco Moccia, Alessandra Fiorio Pla, Dmitry Lim, Francesco Lodola, Andrea Gerbino

**Affiliations:** ^1^ Laboratory of General Physiology, Department of Biology and Biotechnology “L. Spallanzani”, University of Pavia, Pavia, Italy; ^2^ Department of Life Sciences and Systems Biology, University of Torino, Turin, Italy; ^3^ Department of Pharmaceutical Sciences, Università del Piemonte Orientale “Amedeo Avogadro”, Novara, Italy; ^4^ Department of Biotechnology and Biosciences, University of Milan-Bicocca, Milan, Italy; ^5^ Center for Nano Science and Technology @PoliMi, Istituto Italiano di Tecnologia, Milan, Italy; ^6^ Department of Biosciences, Biotechnologies and Environment, University of Bari Aldo Moro, Bari, Italy

**Keywords:** Ca^2+^ signalling, lysosomal Ca^2+^, mitochondria-ER contact sites, TRP channels, non-canonical signalling, optical stimulation

## Abstract

Cytosolic Ca^2+^ signals are organized in complex spatial and temporal patterns that underlie their unique ability to regulate multiple cellular functions. Changes in intracellular Ca^2+^ concentration ([Ca^2+^]_i_) are finely tuned by the concerted interaction of membrane receptors and ion channels that introduce Ca^2+^ into the cytosol, Ca^2+^-dependent sensors and effectors that translate the elevation in [Ca^2+^]_i_ into a biological output, and Ca^2+^-clearing mechanisms that return the [Ca^2+^]_i_ to pre-stimulation levels and prevent cytotoxic Ca^2+^ overload. The assortment of the Ca^2+^ handling machinery varies among different cell types to generate intracellular Ca^2+^ signals that are selectively tailored to subserve specific functions. The advent of novel high-speed, 2D and 3D time-lapse imaging techniques, single-wavelength and genetic Ca^2+^ indicators, as well as the development of novel genetic engineering tools to manipulate single cells and whole animals, has shed novel light on the regulation of cellular activity by the Ca^2+^ handling machinery. A symposium organized within the framework of the 72nd Annual Meeting of the Italian Society of Physiology, held in Bari on 14–16th September 2022, has recently addressed many of the unexpected mechanisms whereby intracellular Ca^2+^ signalling regulates cellular fate in healthy and disease states. Herein, we present a report of this symposium, in which the following emerging topics were discussed: 1) Regulation of water reabsorption in the kidney by lysosomal Ca^2+^ release through Transient Receptor Potential Mucolipin 1 (TRPML1); 2) Endoplasmic reticulum-to-mitochondria Ca^2+^ transfer in Alzheimer’s disease-related astroglial dysfunction; 3) The non-canonical role of TRP Melastatin 8 (TRPM8) as a Rap1A inhibitor in the definition of some cancer hallmarks; and 4) Non-genetic optical stimulation of Ca^2+^ signals in the cardiovascular system.

## 1 Introduction

An increase in intracellular Ca^2+^ concentration ([Ca^2+^]_i_) can operate over a very wide dynamic range to specifically regulate a multitude of cellular functions (Berridge et al., 2003). Neurotransmitter release from presynaptic terminals, as well as insulin exocytosis from pancreatic β-cells, occur within microseconds on the elevation in [Ca^2+^]_i_, while the intracellular Ca^2+^ oscillations that drive gene expression may last for a few hours (Berridge et al., 2003; Clapham, 2007). An additional mechanism that enriches the versatility of intracellular Ca^2+^ signalling is represented by the spatial location of the Ca^2+^ sources, which can be physically coupled to different Ca^2+^-dependent decoders (Berridge et al., 2003; Bagur and Hajnoczky, 2017; Ong et al., 2019; Barak and Parekh, 2020). Environmental cues generate a complex choreography of intracellular Ca^2+^ signals (Berridge et al., 2003; Clapham, 2007), whose spatio-temporal malleability enables one single ion messenger to control as many different functions as fertilization (Moccia et al., 2006), cell cycle (Lim et al., 2003) and proliferation (Faris et al., 2019; Faris et al., 2022), migration (Fiorio Pla et al., 2012; Zuccolo et al., 2018b), differentiation (Maione et al., 2022), contraction (Bers, 2008; Landstrom et al., 2017), metabolism (Patella et al., 2015), angiogenesis (Bernardini et al., 2019; Moccia et al., 2019b; Scarpellino et al., 2020), vasculogenesis (Moccia et al., 2012; Moccia et al., 2013; Zuccolo et al., 2018a), and, more recently, neurovascular coupling (Negri et al., 2021c; Soda et al., 2023). The multifaceted nature of intracellular Ca^2+^ signalling can be further appreciated by recalling that, depending on the Ca^2+^ source and on the Ca^2+^-dependent target, an increase in [Ca^2+^]_i_ may induce opposing cellular responses, e.g., proliferation (Faris et al., 2022) and apoptosis (Astesana et al., 2021; Faris et al., 2023), vascular smooth muscle cell contraction (Knot and Nelson, 1998) and relaxation (Nelson et al., 1995), neuronal depolarization (Menigoz et al., 2016) and hyperpolarization (Tiwari et al., 2018), long-term potentiation (Ezra-Nevo et al., 2018; Soda et al., 2019; Locatelli et al., 2021) and long-term depression (Hirano, 2013). Dysregulation of the sophisticated machinery that orchestrates the Ca^2+^ response to physiological signals can, therefore, trigger or exacerbate a growing list of life-threatening disorders, such as neurodegenerative (Lim et al., 2014; Lim et al., 2021a) and cardiovascular (Venetucci et al., 2012; Moccia et al., 2019a) disorders, severe combined immunodeficiency (SCID) (Vaeth et al., 2020), and cancer (Moccia, 2018; Prevarskaya et al., 2018).

The Ca^2+^ response to environmental cues in non-excitable cells is usually triggered by the phospholipase C-dependent production of inositol-1,4-5-trisphosphate (InsP_3_), which mobilizes Ca^2+^ from what is regarded the most abundant intracellular Ca^2+^ reservoir, namely, the endoplasmic reticulum (ER) (Berridge et al., 2003; Clapham, 2007). InsP_3_ gates the ionotropic InsP_3_ receptors (InsP_3_Rs), which are non-selective cation channel located on ER cisternae, in the presence of a permissive concentration of ambient Ca^2+^ (Prole and Taylor, 2019). Repetitive events of InsP_3_-evoked Ca^2+^ release may be spatially confined to peripheral InsP_3_Rs, which are located in close proximity to plasmalemmal G_q_-Protein Coupled Receptors (G_q_PCRs) (Keebler and Taylor, 2017; Thillaiappan et al., 2017), or can propagate as regenerative Ca^2+^ waves through the mechanism of Ca^2+^-induced Ca^2+^ release (CICR) (Bootman et al., 1997). Ryanodine receptors (RyRs), which represent the main Ca^2+^-releasing channel in the sarcoplasmic reticulum (SR) and may also be present in the ER, support InsP_3_-evoked regenerative Ca^2+^ waves in some, but not all (Moccia et al., 2019b), cell types (Santulli et al., 2018). Depletion of the ER/SR Ca^2+^ content due to cyclic Ca^2+^ extrusion in the extracellular milieu by plasma membrane Ca^2+^-ATPase or Na^+^/Ca^2+^ exchanger (NCX) (Moccia et al., 2002; Berra-Romani et al., 2023) is prevented by the activation of store-operated Ca^2+^ entry (SOCE) (Lewis, 2020; Moccia et al., 2023). SOCE requires the dynamic interplay between Stromal Interaction Molecules 1 and 2 (STIM1 and STIM2, respectively), which serve as sensor of ER Ca^2+^ concentration ([Ca^2+^]_ER_), and the Ca^2+^-selective channels, Orai1-3, on the plasma membrane (Lewis, 2020; Moccia et al., 2023). In excitable cells, membrane depolarization evoked by excitatory synaptic transmission (Locatelli et al., 2021) or spontaneous diastolic depolarization (Eisner et al., 2017) can lead to extracellular Ca^2+^ entry through multiple types of voltage-operated Ca^2+^ channels (VOCCs), which can be followed by CICR through RyRs and/or InsP_3_Rs (Bading, 2013; Eisner et al., 2017). In both excitable and non-excitable cells, extracellular Ca^2+^ entry is further mediated by the Transient Receptor Potential (TRP) family of non-selective cation channels, most of which are polymodal Ca^2+^-permeable channels able to sense chemical, thermal and mechanical signals and thereby execute the most appropriate cellular response (Curcic et al., 2019; Vangeel and Voets, 2019; Diver et al., 2022). The advent of novel high-speed, 2D and 3D time-lapse imaging techniques, single-wavelength and genetic Ca^2+^ indicators, as well as the development of novel genetic engineering tools to manipulate single cells and whole animals, has shed novel light on the regulation of cellular activity by the Ca^2+^ handling machinery (Lim et al., 2016a; Bagur and Hajnoczky, 2017; Tapella et al., 2020; Berra-Romani et al., 2021; Leoni et al., 2021; Longden et al., 2021; Marta et al., 2022). For instance, it has been recognized that ER cisternae may establish dynamic contacts with other intracellular organelles, such as mitochondria (Csordas et al., 2010; Csordas et al., 2018; Bartok et al., 2019; Lim et al., 2021a; Sanchez-Vazquez et al., 2023) and lysosomes (Kilpatrick et al., 2013; Atakpa et al., 2018; Faris et al., 2022), to shape intracellular Ca^2+^ signals. The Ca^2+^-dependent inter-organellar communication between ER and mitochondria has long been known to dictate cellular fate (Loncke et al., 2021; Bonora et al., 2022). We now know that, although both InsP_3_Rs in ER cisternae and mitochondria in the cytosol are quite motile, they can establish temporary interactions at mitochondria-associated ER membranes (MAMs) to increase mitochondrial Ca^2+^ in an InsP_3_-dependent manner and stimulate cellular bioenergetics (Gherardi et al., 2020; Katona et al., 2022). However, stress conditions, such as those that can lead to neurodegenerative disorders, can alter the distance between the ER and mitochondria and, thereby, impair mitochondrial Ca^2+^ uptake and cellular bioenergetics that contributes to cell dysfunction (Lim et al., 2021a; Lim et al., 2023). An unexpected mode of Ca^2+^-dependent inter-organellar communication has also been described at the membrane contact sites between ER and lysosomes (Kilpatrick et al., 2013; Ronco et al., 2015). Herein, the second messenger nicotinic acid adenine dinucleotide phosphate (NAADP), which can also be synthesized upon G_q_PCR or tyrosine kinase receptor (TKR) activation, gates two pore channels (TPCs) to mediate lysosomal Ca^2+^ release and prime ER-embedded InsP_3_Rs for InsP_3_-dependent activation (Patel, 2015; Galione et al., 2023). Lysosomal Ca^2+^ can also be mobilized by TRP Mucolipin 1 (TRPML1), which plays a crucial role in autophagic progression (Medina et al., 2015; Di Paola et al., 2018). TRPML1-mediated Ca^2+^ signals were thought to be confined to the perilysosomal Ca^2+^ space (Medina et al., 2015), but recent studies unexpectedly reported TRPML1-induced global Ca^2+^ signals via the Ca^2+^-dependent recruitment of RyRs and InsP_3_Rs (Kilpatrick et al., 2016; Thakore et al., 2020). An additional dogma that has recently turn into a signalling revolution regards the same operation mode of ion channels. Channel proteins do more than simply conducting biologically relevant ions (Montes de Oca Balderas, 2022). Indeed, emerging evidence indicates that ion channels can signal in a flux-independent mode, thereby widening their potential impact on cell physiology (Borowiec et al., 2014; Vrenken et al., 2016; Chinigo et al., 2020; Pressey and Woodin, 2021; Arcangeli et al., 2023). For instance, the intracellular domains of some VOCCs, i.e., Ca_V_1.2 (Gomez-Ospina et al., 2006) and Ca_V_2.1 (Kordasiewicz et al., 2006), as well as some isoforms of the accessory Ca_V_β subunit (Hibino et al., 2003), can translocate into the nucleus and induce Ca^2+^-independent gene expression. Furthermore, some ionotropic receptors, such as N-methyl-D-aspartate (NMDA) receptors (Montes de Oca Balderas and Aguilera, 2015; Negri et al., 2021a) and type A γ-aminobutyric acid (GABA) receptors (Negri et al., 2022b), can signal an increase in [Ca^2+^]_i_ in a flux-independent manner due to their ability to interact with their corresponding metabotropic receptors. Several members of the TRP superfamily can also function in a non-canonical mode. For instance, TRP Melastatin type 7 (TRPM7) channel promotes most of its effect thought the intrinsic kinase activity that is located within its COOH-terminus (Desai et al., 2012; Faouzi et al., 2017; Cai et al., 2018), whereas TRP Canonical type 1 (TRPC1) does not need to mediate Ca^2+^ to induce proliferation in human umbilical cord vein endothelial cells (Abdullaev et al., 2008). Finally, the versatility of the Ca^2+^ handling machinery has been exploited to design alternative therapeutic avenues for many diseases that are still waiting for an effective treatment. For instance, a light-operated Ca^2+^ permeable channel (LOC) has been generated by introducing plant-derived photosensory domain into a cytoplasmic loop of the Orai1 channel (He et al., 2021). Optogenetic intervention by this novel LOC proved effective to suppress excessive hematopoietic stem cell self-renewal and to alleviate neurodegeneration in a model of amyloidosis (He et al., 2021).

A symposium organized within the framework of the 72nd Annual Meeting of the Italian Society of Physiology, held in Bari on 14–16th September 2022, has recently addressed many of the unexpected mechanisms whereby intracellular Ca^2+^ signalling regulates cellular fate in healthy and disease. The symposium, named “Ca^2+^ signalling: unexpected new roles for the usual suspect”, gathered together four renowned Italian physiologists, who informed a numerous and very interested audience about their novel findings regarding the following topics: 1) the role of TRPML1 in Ca^2+^-mediated water reabsorption in the kidney (Prof. Andrea Gerbino, University of Bari Aldo Moro); 2) the modulation of the ER-mitochondria distance to fuel cellular metabolism in astrocytes and prevent neurodegeneration in Alzheimer’s disease (Prof. Dmitry Lim, University of Piemonte Orientale, Novara); 3) the non-canonical role of TRP Melastatin 8 (TRPM8) in the definition of some cancer hallmarks (Prof. Alessandra Fiorio Pla, University of Turin); and 4) the use of novel light-sensitive organic actuators to stimulate angiogenesis and control cardiac cells pacing (Prof. Francesco Lodola, University of Milan-Bicocca). Herein, we present a full report of the symposium and discuss the implications for the Ca^2+^ signalling field of the novel findings that were presented during each lecture.

## 2 TRPML1 and aquaporin 2: the secret liaison mediated by lysosomal Ca^2+^

Lysosomes are multifunctional organelles: apart from well-defined digestive tasks (Xu and Ren, 2015), lysosomes act as a regulatory hub integrating multiple cues to modulate a wide spectrum of intracellular signaling pathways (Ballabio, 2016). Lysosomal vesicles are emerging as a novel Ca^2+^ reservoir that can finely modulate cellular fate through local or global Ca^2+^ signals (Patel and Cai, 2015; Galione, 2019; Galione et al., 2023). Throughout the whole process, lysosomes can freely diffuse and deliver/reuptake Ca^2+^ in the close proximity of target organelles such as ER, mitochondria and secretory vesicles. Understanding how lysosomes establish the Ca^2+^-dependent cross-talk with surrounding organelles that orchestrate the Ca^2+^ response to physiological cues is crucial to appreciate how defective lysosomal Ca^2+^ signalling underpins life-threatening diseases, such as cancer (Faris et al., 2018), viral infections (Moccia et al., 2021a), hypertension (Negri et al., 2021b) and arrhythmias (Negri et al., 2021b), and lysosomal storage disorders (Kiselyov et al., 2010; Lloyd-Evans et al., 2010; Morgan et al., 2011).

**FIGURE 1:**
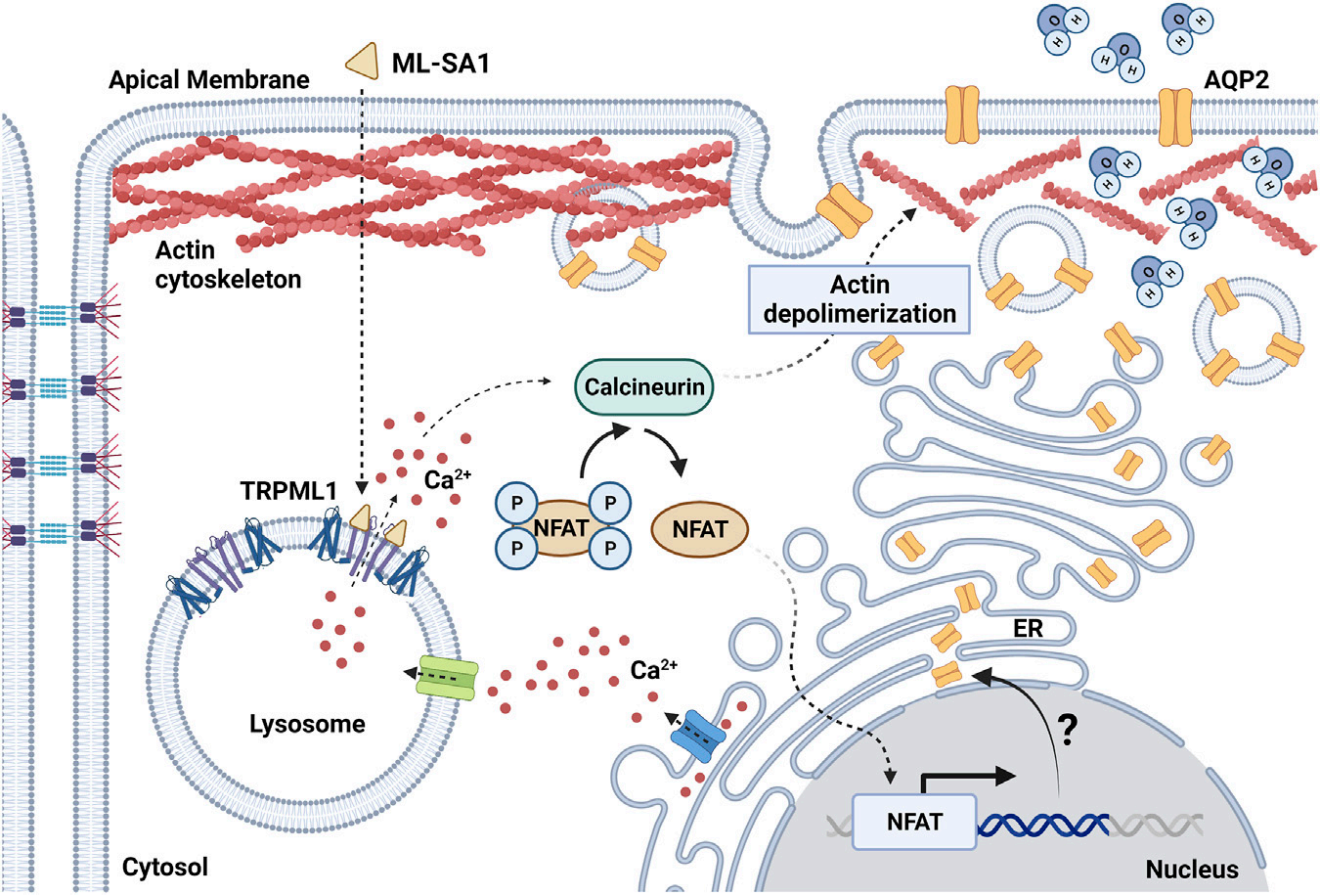
Schematic diagram showing the effect of TRPML1 activation on AQP2-mediated water reabsorption in mouse renal collecting duct cells. ML-SA1 triggers TRPML1-dependent local Ca^2+^ events that are sustained by the endoplasmic reticulum (ER) Ca^2+^ content. Activation of the Ca^2+^/calcineurin/NFAT pathway determines depolymerization of the actin cytoskeleton, thus leading to accumulation of AQP2 at the apical plasma membrane and enhancing water membrane permeability. The putative role of lysosomal Ca^2+^ signaling events as switch for changes in AQP2 expression level through the modulation of the transcriptional activity of NFAT needs further investigation (question mark). Created with BioRender.com (agreement number: FY259UYCKW).

The lysosomal matrix is strongly acidic with a pH of around 4.6 originated by the continuous activity of a vesicular H^+^-proton pump ATPase (V-ATPase) (Xu and Ren, 2015). Lysosomes can actively accumulate large amount of free Ca^2+^ (0.5 mM) through a mechanism that is still highly debated (Yang et al., 2019). Refilling with the Ca^2+^ of the lysosomal matrix could be driven either by a putative H^+^/Ca^2+^ exchanger in a pH-dependent manner (Christensen et al., 2002; Ronco et al., 2015; Melchionda et al., 2016) or by extracellular Ca^2+^ entry through endocytosis or SOCE (Gerasimenko et al., 1998; Sbano et al., 2017). Lysosomal Ca^2+^ can be released into the cytosol through TPCs (Patel, 2015), of which two isoforms exist in mammals (i.e., TPC1 and TPC2), and TRPML1 (Faris et al., 2018). TPCs are gated by NAADP, which can be produced upon G_q_PCR or TKR activation on the plasma membrane, and phosphatidylinositol-3, 5-bisphosphate (PIP_2_) (Patel, 2015; Galione et al., 2023). Intriguingly, planar lysosomal patch-clamp recording showed that NAADP evoked TPC2-mediated currents that were equally mediated by Na^+^ and Ca^2+^, while those gated by PIP_2_ were relatively Na^+^-selective (Gerndt et al., 2020). TPCs can be located at membrane contact sites (MCSs) between lysosomes and ER (Kilpatrick et al., 2017; Faris et al., 2022), where they are physiologically activated by NAADP to release lysosomal Ca^2+^ and evoke global Ca^2+^ signals via Ca^2+^-induced Ca^2+^ release through InsP_3_Rs and/or ryanodine receptors (Patel, 2015; Galione et al., 2023). According to the “trigger-hypothesis” (Galione, 2019; Galione et al., 2023), the InsP_3_-induced Ca^2+^ response to a plethora of extracellular stimuli, including glutamate (Foster et al., 2018; Zuccolo et al., 2019), acetylcholine (Aley et al., 2013), foetal bovine serum (Faris et al., 2019), and vascular endothelial growth factor (VEGF) (Moccia et al., 2021b), is initiated by the NAADP-sensitive lysosomal TPCs. TRPML1 is a non-selective cation permeable channel that mediates lysosomal Ca^2+^, Fe^2+^, and Zn^2+^ release into the cytosol in response by either endogenous agonists, such as phosphatidylinositol 3,5-bisphosphate [PI(3,5)P_2_] (Gan et al., 2022) and reactive oxygen species (ROS) (Zhang et al., 2016) or synthetic ligands, such as ML-SA1 (Kilpatrick et al., 2016). TRPML1 usually mediates local events of Ca^2+^ release that stimulate autophagy by inducing the nuclear translocation of the Ca^2+^-sensitive transcription factor, TFEB (Medina et al., 2015; Di Paola et al., 2018). Furthermore, TRPML1-induced local Ca^2+^ release modulates additional lysosomal functions, including lysosomal exocytosis, membrane trafficking and biogenesis (Di Paola et al., 2018; Medina, 2021). Recent evidence, however, showed that local lysosomal Ca^2+^ release through TRPML1 can also lead to global elevations in [Ca^2+^]_i_ via CICR through InsP_3_Rs (Kilpatrick et al., 2016) or RyRs (Thakore et al., 2020). The Ca^2+^-dependent crosstalk between TRPML1 and ER/SR-resident Ca^2+^-permeable channels is, however, seemingly looser as compared to TPCs. In agreement with this evidence, a recent investigation showed that local Ca^2+^ release events through TRPML1 control water homeostasis in the renal collecting duct (CD, Figure 1) (Scorza et al., 2023).

Facultative water reabsorption in CD cells is finely tuned by a plethora of intracellular signaling mediators and transcription factors (Knepper et al., 2015). Antidiuresis is activated upon the release of the antidiuretic hormone (ADH) by the posterior pituitary gland. Specific binding of ADH with the vasopressin type 2 receptor (V2R), which is localized in principal cells of the CD, stimulates the cAMP/protein kinase A (PKA) axis leading to the apical fusion of the water channel aquaporin 2 (AQP2)-harboring vesicles (Zhao et al., 2023). The rapid apical accumulation of AQP2 boosts water permeability that, in the presence of the strong osmotic gradient in the kidney medulla, is responsible for water reabsorption in the interstitium. The ADH-dependent increase in [Ca^2+^]_i_ is likewise important to enable the proper fusion of AQP2 vesicles with the plasma membrane. Therefore, it does not come as a surprise that Ca^2+^ signaling events can independently influence AQP2 expression and translocation even in the absence of cAMP-mediated cues (Chou et al., 2000; Procino et al., 2015; Mamenko et al., 2016; Tomilin et al., 2019). For instance, the antidiabetic drug rosiglitazone facilitates AQP2 apical accumulation and water reabsorption by inducing massive Ca^2+^ influx upon the specific activation of Transient Receptor Potential Vanilloid 6 (TRPV6) channel (Procino et al., 2015). In addition, a wide variety of TRP channels have been reported in CD cells and CD-derived cultures (Woudenberg-Vrenken et al., 2009). The activation of these channels orchestrates Ca^2+^ responses that are mainly driven by remarkable Ca^2+^ influx often associated with additional Ca^2+^ release from the ER. These robust Ca^2+^ signals can rapidly invade the bulk of the cytosol thus engaging a number of Ca^2+^-dependent molecular effectors localized throughout the cell. Conversely, only scarce information is currently available regarding the role of local Ca^2+^ signals in AQP2-mediated water homeostasis. A recent investigation provided the first evaluation of lysosomal Ca^2+^ signaling events in renal CD cells, which were evoked by either blocking the vacuolar H-ATPase (V-ATPase) with bafilomycin A1 to deplete the lysosomal Ca^2+^ pool (Morgan et al., 2015) or activating TRPML1 with the synthetic agonist ML-SA1 (Kilpatrick et al., 2016) (Figure 1). In CD cells, both lysosomal agonists induced robust and long-lasting cytosolic Ca^2+^ oscillations sustained by tonic ER Ca^2+^ release through InsP_3_Rs but not directly associated to lysosomal Ca^2+^-triggered CICR (Scorza et al., 2023), as widely reported for TPCs (Macgregor et al., 2007; Brailoiu et al., 2009; Kilpatrick et al., 2013; Faris et al., 2019; Moccia et al., 2021b; Faris et al., 2022). This finding strongly suggests that InsP_3_-mediated ER Ca^2+^ release drives lysosomal Ca^2+^ refilling in CD cells. ML-SA1 and bafilomycin A1 differentially modulated AQP2 translocation to the apical membrane and actin polymerization in the cytosol, since only ML-SA1 specifically elicited submaximal water reabsorption in collecting duct cells (Scorza et al., 2023) (Figure 1). Even though ML-SA1 increased water permeability to the same extent as submaximal doses of the cAMP increasing agents forskolin and IBMX, TRPML1 activation was unable to switch on the cAMP/PKA pathway. Currently, the cytosolic Ca^2+^ effectors translating TRPML1-mediated Ca^2+^ release into an increase in AQP2-containining vesicle translocation to the apical membrane remain to be deciphered. However, TRPML1-dependent AQP2 translocation and actin depolymerization were inhibited by blocking the Ca^2+^-dependent phosphatase calcineurin (CaN) with cyclosporine A (Scorza et al., 2023). Intriguingly, CaN is selectively engaged by TRPML1-mediated lysosomal Ca^2+^ release to drive the nuclear translocation of TFEB (Medina et al., 2015), the master regulator of lysosomal function and autophagy (Di Paola et al., 2018; Medina, 2021) (Figure 1). CaN activation tightly bridges lysosomal Ca^2+^ signaling events and water reabsorption by directly dephosphorylating cytoskeletal organizing proteins (cofilin, WAVE-1 and synaptopodin) or eliciting long-lasting transcriptional effects mediated by NFAT (Descazeaud et al., 2012). Therefore, it is reasonable to assume that TRPML1 can regulate water balance by influencing the polymerization state of the actin cytoskeleton thus facilitating the fusion of AQP2-harboring vesicles with the apical plasma membrane (Figure 1). Noteworthy, TRPML1 induced Ca^2+^ events have been associated with fusion of gastric tubulovesicles carrying the H^+^/K^+^-ATPase that pumps H+ into the gastric lumen (Sahoo et al., 2017).

## 3 Ca^2+^ handling at the mitochondria-ER contact sites: role in Alzheimer’s disease-related astroglial dysfunction and beyond

Mitochondrial enzymes and F_0_F_1_ ATP synthase require Ca^2+^ for activation and maintenance of bioenergetic activity and production of ATP. Mitochondria uptake Ca^2+^ with a high affinity directly from juxtaposed InsP_3_Rs located in mitochondria-associated ER membranes (MAMs) (Rizzuto et al., 1993). The morpho-functional complex that holds together interacting ER and mitochondria is referred to as mitochondria-ER contact sites (MERCS) (Herrera-Cruz and Simmen, 2017). Ca^2+^ transfer at MERCS occurs through a complex composed of InsP_3_Rs, voltage-dependent anion channel 1 (VDAC1) and the associated protein Grp75, and then, into mitochondrial matrix, via a low affinity mitochondrial Ca^2+^ uniporter. Besides Ca^2+^ fluxes, MERCS are responsible for a number of key cellular processes, such as lipid and steroid biogenesis, mitochondrial fission and dynamics, autophagosome formation, apoptosis induction, and others (Barazzuol et al., 2021). Disruption of MERCS has been observed in several neurodegenerative diseases, including Parkinson’s disease, amyotrophic lateral sclerosis and Alzheimer’s disease (AD) (Paillusson et al., 2016; Area-Gomez and Schon, 2017; Lim et al., 2021a; Leal and Martins, 2021). In AD, a strengthening of the interaction between ER and mitochondria has been found in human brains and in animal and cellular AD models (Lim et al., 2021a). Although such increase has been associated with mitochondrial dysfunction and with aberrant processing of amyloid precursor protein (APP), mechanistic aspects MERCS alterations and cause-effect relationships with AD-related cellular pathology remain poorly understood (Lim et al., 2021a; Lim et al., 2023).

AD, a major, yet uncurable, age-related neurological disorder, has a long-lasting pathogenesis with poorly characterized preclinical and prodromal phases. Cellular dysfunctions, such as alterations of protein synthesis and degradation with associated accumulation of misfolded/aggregated proteins, mitochondrial dysfunction with concomitant bioenergetic deficit and oxidative stress, and derangement of Ca^2+^ homeostasis and signalling, represent early signs of AD pathology (De Strooper and Karran, 2016). Yet, these dysfunctions have mainly been studied and interpreted from the point of view of neuronal pathology, while alterations in glial cells, specifically in astrocytes, have been largely overlooked (Verkhratsky et al., 2019; Merlo et al., 2021). Astrocytes are homeostatic and supportive cells in the central nervous system (CNS), which warrant correct development, function and adaptation of neurons and other cells in the CNS to activity and stress (Verkhratsky and Nedergaard, 2016; Santello et al., 2019; Tapella et al., 2020). They participate in formation of morpho-functional units in the brain, such as blood-brain barrier (BBB) and neurovascular unit (Schaeffer and Iadecola, 2021), and are responsible for metabolic, structural and functional support to neurons. In AD pathogenesis, astrocytes undergo complex biphasic alterations, first becoming asthenic and atrophic, to turn to hypertrophy and reactivity at later AD stages in concomitance with the development of senile plaques and neurofibrillary tangles accompanied by remodelling of astrocytic Ca^2+^ signalling (Lim et al., 2014; Lim et al., 2016b; Verkhratsky et al., 2019). Reactive astrocytes, in association with microglial cells, participate in the development of neuroinflammatory reaction. During these transformations, astrocytes lose their homeostatic and defensive functions and leave neurons to suffer damage, lose synaptic connectivity and die. Little is known about astrocytic cell pathology during early AD pathogenesis.

Unexpectedly, recent findings suggest that the alterations of MERCS and ER-mitochondrial Ca^2+^ transport may be responsible for a number of cellular dysfunctions, which may explain the loss of homeostatic function by AD astrocytes. These studies took advantage of a novel model of immortalized hippocampal astrocytes from 3xTg mouse model of AD, which faithfully reproduce transcriptional and functional alterations of primary AD astrocytes (Ruffinatti et al., 2018; Rocchio et al., 2019). Moreover, their produce and release β-amyloid peptide and have impaired autophagic and proteasomal protein degradation, which are signs of early cellular dysfunction in AD (Gong et al., 2023). Immortalized WT and 3xTg-AD astrocytes, referred to as WT-iAstro and 3Tg-iAstro, represent versatile and easy-to-handle astrocytic AD model, well suited for comprehensive investigation from single cell imaging and transfection to omics analyses and sub-cellular fractionation requiring large amount of material (Tapella et al., 2023). First, it was assessed whether 3Tg-iAstro present mitochondrial alterations characteristic for AD cells. 3Tg-iAstro cells have a lower basal mitochondrial respiration and severely impaired respiratory reserve, significantly lower mitochondrial ATP production and significantly higher mitochondrial ROS. Glycolytic activity was also impaired in 3Tg-iAstro compared with WT-iAstro cells. This was in line with recent reports on AD-derived human iPSC-differentiated astrocytes (Oksanen et al., 2017; Oksanen et al., 2019). Proteomics analysis on isolated mitochondria and associated ER membranes were also conducted. Surprisingly, differentially expressed proteins were found to be mainly responsible for ER functions and ribosomal proteins synthesis (Dematteis et al., 2020). Validation of these results showed that 3Tg-iAstro cells presented a lower rate of basal protein synthesis and low-grade chronic ER stress accompanied by an increased phosphorylation of eukaryotic initiation factor 2α (p-eIF2α). Gong et al. (2023) found that proteasomal and autophagic activities are impaired in 3Tg-iAstro cells. Moreover, 3Tg-iAstro, but not WT-iAstro cells, were unable to promote the formation of the bidimensional tubular network, which is the *in vitro* surrogate of *in vivo blood* vessel formation (Balbi et al., 2019; Balducci et al., 2021), in an *in vitro* astrocyte/pericyte/endothelial 3D co-culture due to a loss of secreted factors, thereby suggesting the impairment of key homeostatic functions (Tapella et al., 2022). These alterations were also found in hippocampus of 3xTg-AD mice *in vivo* (Tapella et al., 2022).

Next, it was investigated if 3Tg-iAstro presented alterations of Ca^2+^ homeostasis (Lim et al., 2021b). A significant increase of steady-state ER Ca^2+^ level and higher ATP-induced Ca^2+^ signals in the cytosolic compartment, indicating a higher Ca^2+^ ER load and higher InsP_3_R-mediated Ca^2+^ release, were reported. This was in accord with previous reports (Grolla et al., 2013a; Grolla et al., 2013b; Lim et al., 2013; Ronco et al., 2014). However, unexpectedly, ATP-induced Ca^2+^ transients, measured in mitochondrial matrix, were significantly lower in 3Tg-iAstro compared with WT-iAstro cells, indicating on the alterations with ER-mitochondrial Ca^2+^ transport. This was in line with the increased ER-mitochondrial interaction at a distance of 8–10 nm, which we have demonstrated using a split-GFP ER-mitochondrial contact site sensor (SPLICS) (Cieri et al., 2018; Dematteis et al., 2020). To investigate if the increased ER-mitochondrial interaction and the impaired mitochondrial Ca^2+^ signals were responsible for alterations of cellular proteostasis, an artificial linker that fixes the ER and the outer mitochondrial membrane at a short distance of about 10 nm was overexpressed in WT-iAstro cells, thereby reproducing MERCS and Ca^2+^ alterations found in 3Tg-iAstro cells. Strikingly, fixing MERCS at 10 nm reproduced the impairment of ribosomal protein synthesis and increased p-eIF2α levels. Moreover, as reported for 3Tg-iAstro cells, WT-iAstro cells overexpressing 10 nm linker were unable to support tubulogenesis *in vitro* in 3D co-culture with pericytes and endothelial cells (Tapella et al., 2022).

**FIGURE 2:**
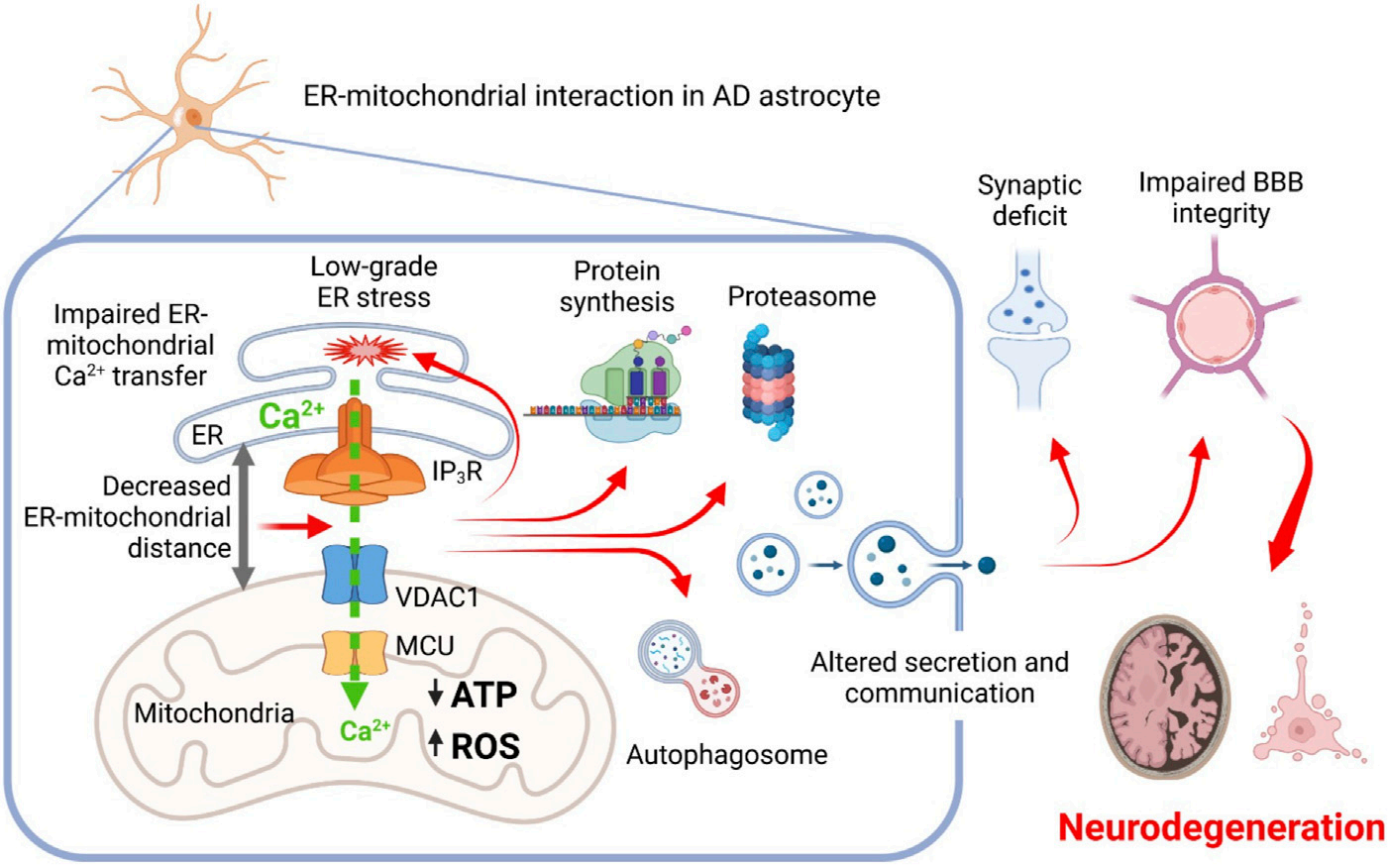
Proposed scheme relationships between AD-related mutations, mitochondrial-ER interaction, mitochondrial and ER Ca^2+^ signaling, and cellular dysfunctions in astrocytes. Altered ER-mitochondrial interaction impairs ER-mitochondrial Ca^2**+**^ transfer, resulting in mitochondrial bioenergetic deficit and increased production of ROS, induction of a low-grade chronic ER stress and derangement of proteins synthesis and degradation. Cellular dysproteostasis results in an impaired secretion of factors including adhesion molecules, components of extracellular matrix, pro-neurogenic and neuroprotective molecules. Altogether, this impairs homeostatic and signaling activity of AD astrocytes eventually leading to impairment of synaptic functions, blood-brain barrier integrity and to development of neurodegeneration.

Taken together, these results provide proof of principle that the shortening of ER-mitochondrial distance, observed in AD, may be causative for a number of cellular AD-related alterations. Furthermore, our results suggest that the altered MERCS function in AD astrocytes may result in impairment of CNS homeostasis, BBB and neuronal dysfunction (Figure 2). Further experiments are necessary to elucidate molecular mechanisms of MERCS dysfunction and dissect the role of impaired ER-mitochondrial Ca^2+^ transfer in AD pathogenesis.

## 4 Non-canonical role of TRP Melastatin 8 (TRPM8) in the definition of some cancer hallmark

TRPM8 is a member of the TRP family primarily known for its classical cold receptor function in sensory neurons required for cold thermal transduction and response as well as pain sensation in mammals (McKemy et al., 2002; Madrid et al., 2006; Dhaka et al., 2008; Knowlton et al., 2013). The first identified “full-length” isoform of TRPM8 consists of a homotetrameric protein of 1,104 amino acid (128 kDa) organized into six hydrophobic transmembrane α-helices (S1-S6) with a transmembrane loop between S5 and S6, and cytosolic tetrameric coiled-coil COOH-terminal domain (C-term) and a large hydrophilic NH_2_-terminal domain (N-term) containing ‘TRPM homology regions’ (MHR) involved in channel assembly and trafficking (Kraft and Harteneck, 2005; Fujiwara and Minor, 2008; Yin et al., 2018). The voltage sensor-like domain (VSLD) is defined by the first 4 TM helices (S1-S4) and also contains the binding sites for menthol and icilin at the cavity formed with the TRP domain (Bandell et al., 2006; Yin et al., 2019). The pore module of TRPM8 is, instead, formed by the last 2 TM helices (S5-S6) and it is characterized by a highly conserved hydrophobic region and a conserved aspartate residue, responsible for ion selectivity (P_Ca_/P_Na_ = 3.3) (Zholos et al., 2011). Interestingly, this full length TRPM8 is mainly localized in the plasma membrane but is also partly present at the ER level where it functions by releasing Ca^2+^ form the store (Chinigo et al., 2022).

Beside this well know role in thermal transduction, the human *TRPM8* gene was first identified and cloned from prostate tissues and described as a new prostate-specific gene due to the peculiar expression pattern shown during prostate cancer (PCa) progression (Tsavaler et al., 2001). In particular, TRPM8 is upregulated in benign hyperplasia (BPH) and during the early androgen-dependent stages of PCa, and then downregulated in the more advanced androgen-independent metastatic stages of the tumor. Consistent with its unique deregulation during PCa progression, alterations in TRPM8 channel activity have been linked to several cancer hallmarks, including tumor cell proliferation and survival, cell migration, and angiogenesis (Alaimo et al., 2020; Grolez et al., 2022).

**FIGURE 3:**
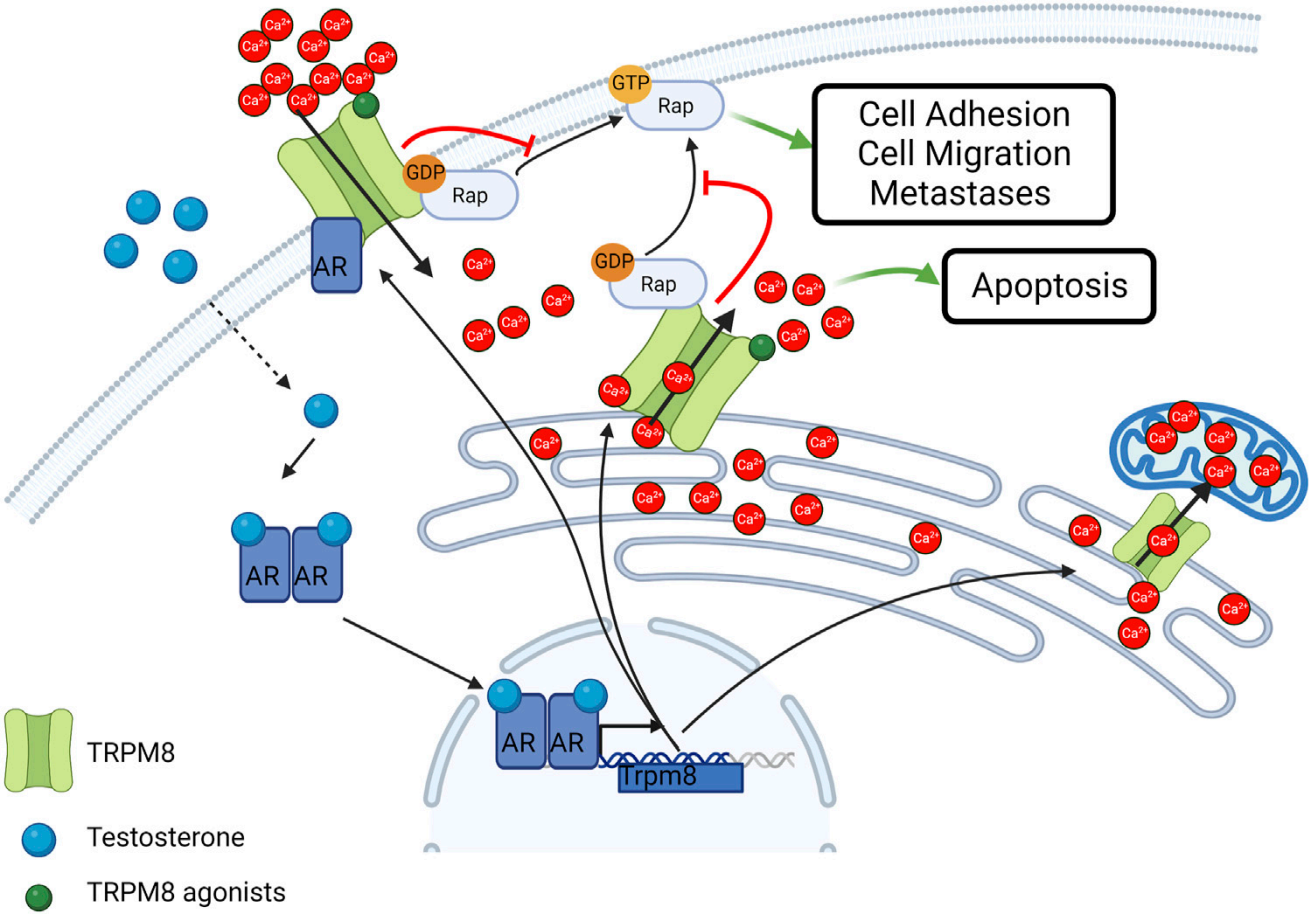
Schematic representation of TRPM8 subcellular localization and activity in cancer cells. TRPM8 Full length isoform localizes at the plasma membrane and is subjected to androgen regulation. Smaller isoforms typically localize in the ER and mediate Ca^2+^ release in the cytosol or Ca^2+^ transfer in the mitochondria. TRPM8 also act independently from its channel activity as an inhibitor of the small GTPase Rap1A thus inhibiting cell adhesion and migration. Created with BioRender.com (agreement number: AT259UYHZ3).

However, the impact of TRPM8 in the development and progression of PCa is subject to complex modulation mechanisms that also underlie the expression of different isoforms with distinct subcellular localization and activity depending on tumor stage and androgen sensitivity. Indeed, the expression of the full-length isoform of TRPM8 located on the plasma membrane (TRPM8_PM_) is highly subject to androgen regulation and thus is significantly downregulated in androgen deprivation and androgen receptor (AR) loss during the late androgen-independent phase of PCa (Zhang and Barritt, 2004; Bidaux et al., 2005; Grolez et al., 2019). This regulation occurs through both genomic and non-genomic mechanisms involving the AR (Figure 3) (Bidaux et al., 2005; Grolez et al., 2019). As regarding in particular the non-genomic action, the role of AR-TRPM8 interaction is tightly regulated by testosterone in a dose-dependent manner: low doses of testosterone (10 nM) are associated with AR-TRPM8 localization at the level of lipid rafts and a significant inhibition of TRPM8 activity which in turn lead to an increase in cell motility as compared with the absence of testosterone; on the other hand, high doses of testosterone (100 nM) lead to a decrease of TRPM8-AR interaction thus reverting the inhibitory effect of AR on TRPM8 activity (Grolez et al., 2019). This loss of interaction and delocalization of TRPM8 outside of lipid rafts, significantly increases prostate cancer cell motility (Grolez et al., 2019).

Beside the role of the full-length TRPM, during the transition from androgen-dependent to androgen-independent phases of PCa, through an alternative splicing mechanism, the “full-length” isoform of TRPM8 gives way to a shorter isoform with typical ER localization, known as TRPM8_ER_ (Bidaux et al., 2007). The TRPM8_ER_ isoform, being able to directly release ER Ca^2+^ and thereby activate SOCE on the plasma membrane, is mainly involved in the control of Ca^2+^-dependent pro-apoptotic mechanisms (Figure 3) (Thebault et al., 2005; Prevarskaya et al., 2007). Interestingly, the pro-apoptotic role of TRPM8 has also been confirmed in PCa cells treated with sub-lethal doses of radio, hormonal, or chemo therapies (Alaimo et al., 2020; Genovesi et al., 2022). Furthermore, other isoforms of the channel have been identified to date in the prostate. A functional TRPM8_ER_ characterized by only 4 rather than 7 transmembrane domains (TMDs) has been identified and characterized as a mediator of the Ca^2+^ transfer from the ER to the mitochondria in PCa epithelial cells (Figure 2) (Bidaux et al., 2018), while short non-channel TRPM8 isoforms (sM8s) with ubiquitous cytosolic localization in PCa were found to exert antagonist functions towards the full-length isoform (Peng et al., 2015; Bidaux et al., 2016). sM8s are a first example of non-channel function of TRPM8 that influences cell behavior independently of pore function and Ca^2+^ mobilization (Fernandez et al., 2012). Therefore, the growth of primary PCa as a result of the equilibrium between proliferation and apoptosis may depend on the relative expression levels of the different TRPM8 isoforms with channel and non-channel functioning.

In addition, TRPM8 regulates cell migration through both Ca^2+^-dependent and Ca^2+^-independent mechanisms. TRPM8-mediated Ca^2+^ signals induce an increase in the expression and activity of some proteins that are crucial in the epithelial-to-mesenchymal transition (EMT), in focal adhesion dynamics and consequently in the control of cell adhesion and migration (Noren et al., 2000; Millar et al., 2017). In particular, Cdc42, Rac1, ERK, and FAK are stimulated in a Ca^2+^-dependent manner by TRPM8 activity in PCa cells (Yang et al., 2009; Zhu et al., 2011; Wang et al., 2012; Grolez et al., 2022). On the other hand, the involvement of TRPM8 in the migratory machinery goes beyond its channel function. Indeed, a novel facet of TRPM8 as an inhibitor of the small GTPase Rap1A that is completely independent of its cation channel activity has recently been unveiled (Figure 3) (Genova et al., 2017; Chinigo et al., 2022). More specifically, a direct physical interaction between TRPM8 and Rap1A has been characterized in both PCa-derived endothelial cells and epithelial PCa cells (Genova et al., 2017; Chinigo et al., 2022). The interaction site is located on the NH_2_-terminus of the channel and involves the glutamate 207 and the tyrosine 240, which directly interact with some residues (including tyrosine 32) located within the switch I region of Rap1A, responsible for the transition from the inactive to the active form of the small GTPase (Chinigo et al., 2022). Indeed, Rap1A, as a small GTPase, co-exists in two different forms: an active form when bound to GTP and an inactive form when bound to the GDP (Vetter and Wittinghofer, 2001). Specific guanine exchange factors (GEFs) catalyze the exchange between GDP and GTP thereby inducing small GTPase activation, which normally results in the promotion of cell adhesion through the activation of the β1-integrin signaling at the plasma membrane (Chrzanowska-Wodnicka et al., 2008; Boettner and Van Aelst, 2009; Carmona et al., 2009; Cherfils and Zeghouf, 2013). Recent work demonstrated that TRPM8 intracellularly binds Rap1A mainly at the ER in its inactive form, thus hindering its translocation to the plasma membrane and its subsequent activation (Genova et al., 2017; Chinigo et al., 2022). This mechanism results in the inhibition of cell adhesion and migration in PCa-derived endothelial cells and in epithelial PCa cells, thus making TRPM8 an appealing candidate to block both tumor invasiveness and angiogenesis (Genova et al., 2017; Chinigo et al., 2022). Although TRPM8 expression is sufficient to exert these functional effects, stimulation with TRPM8 agonists, such as icilin and WS12, further potentiates these effects not only by recruiting Ca^2+^-dependent pathways, such as Cdc42, Rac1, ERK, and FAK, but also by probably promoting TRPM8-Rap1 interaction. This could be explained by global conformational rearrangements triggered by agonist binding in the TRPM8 TMDs that are propagated to the cytosolic domain where interaction with Rap1A occurs (Yin et al., 2018; Yin et al., 2019). Rap1A is not the only GTPase involved in the TRPM8 interactome. Indeed, TRPM8 was found to interact with the inactive form of the G-protein subunit Gαq, which leads to the inhibition of TRPM8 gating and, in turn, may be subject to TRPM8-mediated metabotropic regulation (Klasen et al., 2012; Zhang et al., 2012). These data fit into the broader context of a bidirectional close interplay between TRP channels and small GTPases at all stages of the metastatic cascade through both Ca^2+^-dependent and Ca^2+^-independent pathways (Chinigo et al., 2020).

All these recent mechanistic findings on TRPM8 provide new insights for the development of innovative and effective tools targeting TRPM8 to block PCa progression and improve the prognosis of the currently incurable metastatic castration-resistant prostate cancer (mCRPC) phenotypes. In addition to supporting a potential use of TRPM8 in anti-tumor therapy as a dual target to simultaneously counteract metastatic dissemination and angiogenesis, they also shed new light on the possibility of using TRP channels as targets for the development of peptidomimetics in cancer therapy. In fact, the administration of therapeutic peptide mimicking the channel or part of its structure would further reduce any side effects associated with the wide tissue distribution of TRP channels and the multitude of intracellular signalling pathways regulated by them, directly targeting a specific protein-protein interaction and consequently impairing only its associated cellular pathways (Mabonga and Kappo, 2019; Tsagareli and Nozadze, 2020). As to TRPM8-Rap1A interaction, the applicability of a peptide that reproduces the N-terminus of the channel in patients in androgen-independent late stages of PCa seems to be further supported by the fact that none of the residues involved in this interaction were mutated in the analyzed patient cohorts (Chinigo et al., 2022). Of note, validation of TRPM8-Rap1A interaction in more than 1 cell line (Genova et al., 2017; Chinigo et al., 2022), including prostate, breast, and cervical cancer cells as well as endothelial cells, suggests a broader spectrum of action of TRPM8 as an inhibitor of Rap1, albeit with a different impact in terms of control of cell adhesion and migration according to the cell type. Therefore, this protein-protein interaction could prove to be an interesting target in the treatment of a much wider range of pathologies.

## 5 Non-genetic light stimulation of Ca^2+^ signals in cardiovascular research: methodology and possible applications

The idea to use light to trigger specific biological pathways, including Ca^2+^ signalling, represents one of the most fascinating insights in modern science (Lodola and Moccia, 2022). In recent years, photostimulation of cells and living systems has received great interest from the scientific community due to several unique advantages. Indeed, light is a minimally invasive biophysical tool that can overcome the limitations of more conventional stimulation approaches based on electrical, chemical, mechanical, or magnetic cues (i.e., limited spatial and temporal resolution) (Di Maria et al., 2018). The potential revolutionary role of light has been originally suggested by Sir Francis Crick. The Nobel Prize for Physiology or Medicine, discussing the need to achieve a selective control of individual neurons to understand the complexity of the brain, asserted that “*The ideal signal would be light, probably at an infrared wavelength to allow the light to penetrate far enough. This seems rather farfetched, but it is conceivable that molecular biologists could engineer a particular cell type to be sensitive to light in this way*” (Crick, 1999). This revolutionary concept become reality few years later with the implementation of Optogenetics, which consists in the expression of light-sensitive ion channels into the cellular plasma membrane to control the activity of neurons or other cell types with light (Deisseroth, 2011). However, the standard method to deliver the light-sensitive sensors-actuators to the target cells membrane impinges on viral constructs and this, combined with the fact that the exogenous proteins are isolated from very distant species (i.e., bacteria, algae, or unicellular fungi), open a series of issues in the therapeutic translatability of the approach.

An alternative strategy to still preserve the advantages of optical stimulation, but avoiding genetic modification, relies on the use of photosensitive transducers (Di Maria et al., 2018; Hopkins et al., 2019). The foundation of this approach is built on the convergence of various cutting-edge expertise ranging from biology, material science and photonics. In recent years, both inorganic and organic semiconductors have been used with excellent results (Di Maria et al., 2018; Hopkins et al., 2019). In particular, the organic one has aroused considerable interest within the scientific community due to their unique characteristics. In fact, these materials support both ionic and charge transfer, are soft and conformable, cost-effective and solution processable, but most importantly their absorption range is in the visible region, and they present a high biocompatibility, thus proving capable of interfacing with living matter to transduce light into a biological signal. Regioregular polymer poly(3-hexylthiophene-2,5-diyl), referred as P3HT, is probably the workhorse material among the organic semiconductors and the widely studied for biological purposes (Antognazza et al., 2015; Di Maria et al., 2018; Moccia et al., 2020).

The main photophysical mechanisms that occur at the polymer/cell interface could be capacitive, electrochemical, or thermally mediated. These phenomena in turn generate different cellular response. For example, at the cellular level, planar P3HT has been proven effective in the modulation of the membrane potential of non-excitable cells (i.e., HEK-293 cells and astrocytes) up to the optical stimulation/silencing of neuronal firing (Ghezzi et al., 2011; Benfenati et al., 2014; Antognazza et al., 2015; Feyen et al., 2016; Di Maria et al., 2018). Notably, its efficacy is not limited to *in vitro* applications. Indeed P3HT-based hybrid interfaces (Ghezzi et al., 2013; Antognazza et al., 2016; Maya-Vetencourt et al., 2017), and more recently also nanoparticles (Maya-Vetencourt et al., 2020), were also shown to restore light-sensitivity and visual acuity in animal models of retinal degeneration evidencing novel potential biomedical implications of conjugated polymers.

**FIGURE 4:**
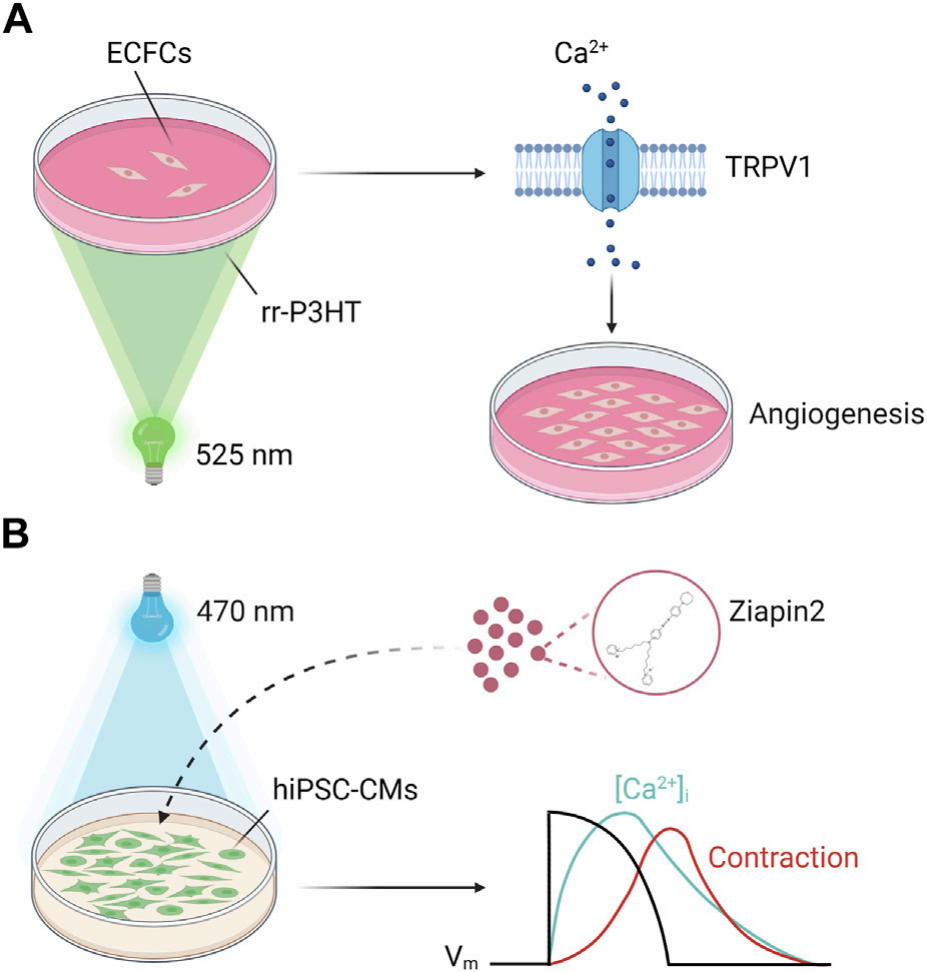
Geneless light stimulation of Ca^2+^ signals in cardiac cells. **(A)** Polymer-mediated optical excitation induces a robust enhancement of proliferation and bidimensional tube formation in ECFCs seeded on top of P3HT thin films (λ = 520 nm). ECFC modulation in ECFCs requires TRPV1 activation on the plasma membrane, which in turn mediates extracellular Ca^2+^ entry to engage a NF-kB-dependent gene expression program. **(B)** Ziapin2 internalizes into the plasma membrane of human induced pluripotent stem cell-derived cardiomyocytes (hiPSC-CMs). Upon photoexcitation (λ = 470 nm) the molecule isomerizes, changing hiPSC-CMs membrane capacitance. This triggers action potential generation and consequently modulates the “excitation-contraction coupling” process at a whole extent opening a new way towards hybrid soft robotics and heart disease therapies. Adapted from (Vurro et al., 2023a). Created with BioRender.com (agreement number: RO259UYLP6).

The modulation of cellular fate via electrochemical and/or thermal signals could be achieved by modulation of [Ca^2+^]_i_ (Bossio et al., 2018; Moccia et al., 2022). Recently, it has been demonstrated that P3HT photoexcitation led to the activation of the non-selective cation channel Transient Receptor Potential Vanilloid 1 (TRPV1) channel (Lodola et al., 2017b; Moccia et al., 2020; Moccia et al., 2022). TRPV1 is a non-selective cation channel that can integrate a variety of extracellular cues (Moccia et al., 2020; Moccia et al., 2022), including an increase in ROS (Guarini et al., 2012), an increase in temperature >40 °C (Caterina et al., 1997), and by a reduction in extracellular pH (Jordt et al., 2000). In accord, P3HT photoexcitation can stimulate TRPV1-mediated membrane depolarization via the local increase in temperature and ROS concentration at the interface between PH3T thin films and cell membrane (Lodola et al., 2017b). Further studies showed that optical excitation of P3HT thin films induced intracellular Ca^2+^ oscillations in human circulating endothelial colony forming cells ECFCs) (Negri et al., 2022a), a truly endothelial progenitor population that is mobilized in peripheral circulation upon an ischemic insult to regenerate the damaged vascular networks (Moccia et al., 2018). TRPV1-mediated Ca^2+^ signals were mainly elicited by local ROS generation and were supported by InsP_3_-induced ER Ca^2+^ release and SOCE (Negri et al., 2022a). Of note, light-induced intracellular Ca^2+^ oscillations were reminiscent of the repetitive Ca^2+^ spikes whereby vascular endothelial growth factor (Dragoni et al., 2011; Dragoni et al., 2015; Lodola et al., 2017a) and the human amniotic fluid stem cell secretome (Balducci et al., 2021) induce the nuclear translocation of NF-κB to stimulate ECFC proliferation and tube formation. In agreement with these observations, optical excitation of P3HT thin films was found to boost ECFC pro-angiogenic activity by activating TRPV1 and thereby promoting a NF-κB-dependent gene expression program (Figure 4A) (Lodola et al., 2019a). These findings pave the way towards the use of these materials as a reliable tool for precise and reversible optically-driven modulation of ECFC physiological activity (Zhang et al., 2014; Lodola et al., 2019a; Moccia et al., 2020; Moccia et al., 2022).

The same interface has been applied also to optical increase the contractile activity of human induced pluripotent stem cell-derived cardiomyocytes (hiPSC-CMs), a process where Ca^2+^ is the actual coupling between excitation occurring in the sarcolemma and the onset of mechanical contraction (Bers and Guo, 2005). Although in this experimental setting the physical process was photothermal, P3HT still presents advantages over more traditional stimulation methods, thereby opening interesting perspectives for the control of cardiac pacing (Lodola et al., 2019b).

Within this context, an alternative approach involves photochromic compounds (Wang and Li, 2018). These organic molecules undergo reversible transformation between two metastable states following the absorption of an electromagnetic radiation and provide a conceptually simple and convenient way to control cellular activity. Indeed, photoswitches can bind covalently to ion channels/receptors or be targeted directly to the plasma membrane bilayer, thus modifying, upon photoisomerization, the ion channel dynamics and/or the electrical properties of the membrane (Gorostiza and Isacoff, 2008; Izquierdo-Serra et al., 2016; Leippe et al., 2017). Recently, a newly synthetized amphiphilic azobenzene-based photo-transducer (Ziapin2), successfully tested in bacteria, HEK-293 cells and neurons (Paterno et al., 2020a; Paterno et al., 2020b; DiFrancesco et al., 2020; Magni et al., 2022), has been used as a non-invasive optical tool to trigger hiPSC-CMs contraction behavior (Vurro et al., 2023a). Thanks to its peculiar chemical properties Ziapin2 has the capability to dwell within the hiPSC-CMs sarcolemma. In this environment the molecule photoisomerization induces a heatless mechanical perturbation upon millisecond pulse of visible light that leads to a dynamic modulation of membrane capacitance. This change in the passive electrical property of the cell results in a transient hyperpolarization followed by a delayed depolarization able to elicit an action potential. The electrical activity correlates with changes in Ca^2+^ dynamics and ultimately with an increase in the contraction rate (Figure 4B). The photopacing efficacy of the approach has been further extended to a cardiac microphysiological model that mimics the cellular organization and substrate mechanical properties of native cardiac tissue (Vurro et al., 2023b), thus proving that Ziapin2 could be a viable tool for the modulation of the excitation-contraction coupling with a precise spatial and temporal punctuality.

## 6 Conclusion

The Symposium “Ca^2+^ signalling: unexpected new roles for the usual suspect” has been one of the most attended events of the 72nd Annual Meeting of the Italian Society of Physiology. In our opinion, this was not only due to the widespread function of the Ca^2+^ handling machinery, which plays fundamental and diversified roles in human physiology that could of course gather vast interest by the audience. We believe that the Symposium gathered such a large audience since it aimed at a presenting one of the oldest signalling messengers known, i.e., Ca^2+^, from a novel perspective. It is now clear that the Ca^2+^ handling machinery is no longer limited, to quote a few paradigmatic examples, to intracellular Ca^2+^ stores that exclusively located in the ER or to voltage-gated Ca^2+^ channels and ligand-gated channels on the plasma membrane. Lysosomes and mitochondria are also crucial to shape the physiological Ca^2+^ response to extracellular cues by, respectively, amplifying, or modulating ER Ca^2+^ release. Altering this delicate balance of inter-organellar Ca^2+^ fluxes can lead to life-threatening disorders, such as AD, cancer, and lysosomal storage disorders, and many more are likely to be discovered in the next future. The non-canonical function of ion channels, exemplified by TRPM8-Rap1A interaction, represents another revolutionary field of research showing that classical omics technologies, such as single-cell RNA sequencing or mass spectrometry, need to be integrated by a physiological approach to truly understand the signalling mode of a channel transcript/protein. These emerging pieces of information on the heterogeneity and versatility of the Ca^2+^ handling machinery can be exploited to design alternative strategies to selectively rescue the function of diseased cells by combining novel nanotechnologies with a proper knowledge of molecular physiology. Due to its polymodal nature, TRPV1 is certainly the best molecular switch to translate optical stimulation of photosensitive conjugated polymers into a biologically relevant signal. But other candidates presenting similar sensitivity to heat and ROS, such as TRP Ankyrin 1, are likely to be rapidly integrated in the arsenal of Ca^2+^-permeable channels that could be probed for their therapeutic potential. In conclusion, this Symposium, which also engendered may fruitful discussions and opened the way to new collaborations among the participants (including many foreigner guests), confirmed that Italian Physiology is at the forefront of research in Ca^2+^ signalling, as also proven by many other oral and poster presentations of the meeting (Martinotti et al., 2019; Nesher et al., 2019; Badone et al., 2021; Lazzarini et al., 2022; Michelucci et al., 2022; Sforna et al., 2022; Arici et al., 2023; Lia et al., 2023).
